# Hsp90 inhibitor is a good candidate for effective combination therapy with carbon ions

**DOI:** 10.1093/jrr/rrt168

**Published:** 2014-03

**Authors:** Ryuichi Okayasu, Hirokazu Hirakawa, Aya Masaoka, Miho Noguchi, Ryoichi Hirayama, Huizi Li, Yoshitaka Matsumoto, Akira Fujimori

**Affiliations:** 1 National Institute of Radiological Sciences, Chiba, Japan; 2 Japan Atomic Energy Agency, Tokaimura, Japan; 3 Chiba University, Chiba, Japan

**Keywords:** Hsp90 inhibitor, carbon ions, radio-sensitization, 17AAG, PU-H71

## Abstract

**Purpose/Background:** To further improve the effectiveness of heavy ion radio-therapy, we studied the biology of a combined treatment using Hsp 90 inhibitor and carbon ion irradiation. We have previously reported that an Hsp90 inhibitor 17AAG can be an effective radio-sensitizer with X-rays for certain human tumor cells, while normal cells were not sensitized by this drug [ 1]. The underlying mechanism for this was demonstrated to be inhibition of DNA double-strand break (DSB) repair by 17AAG; particularly, homologous recombination repair (HRR) pathway was shown to be affected by this agent. *In vivo* mouse xenograft study also indicated a better tumor control with the combined treatment when compared with X-ray treatment alone.

**Materials and Methods:** Cell lines used were SQ5 human lung cancer cells and HFL III normal human fibroblasts as control. For irradiation, X-rays and 290 MeV/n carbon ions were used at 50–70 keV/µm LET setting. As Hsp90 inhibitors, 17AAG and PU-H71 were used for pre-irradiation treatment for 24 h. Colony formation assay was used for radiation sensitivity studies. Repair of DNA DSBs was measured by constant field gel electrophoresis, and the appearance/disappearance of Rad51 foci was analyzed for HRR efficiency. For IRB-approved mouse xenograft study, BALB/c nu/nu mice were used to implant SQ5 cells for local irradiation.

**Results/Discussion:** SQ5 tumor cells were better controlled with the combination of 17 AAG and carbon ion irradiation *in vitro* and *in vivo* xenograft model when compared with carbon irradiation alone. The cause of this radio-sensitizing effect seems to come from inhibition of repair of DNA DSBs by 17AAG as in X-rays. Likewise, HRR pathway was affected by addition of 17AAG in carbon irradiated tumor cells. The effect of 17AAG pre-treatment is shown below where the appearance of an HRR key protein Rad51 was significantly delayed after carbon ion irradiation in the drug-treated samples when compared with samples with carbon alone (Fig. 1).

We also started to investigate PU-H71 with carbon ion irradiation *in vitro* and *in vivo*. PU-H71 alone is currently under phase I clinical trial. Our data indicate that PU-H71 pre-treatment also showed a significant radio-sensitization in SQ5 human lung tumor cells exposed to carbon ions as well as to X-rays. As shown with 17AAG, normal human cells were not significantly affected with this drug. PU-H71 also seems to affect repair of radiation-induced DSBs. Further mechanism studies and *in vivo* experiments are currently underway.

**Conclusion:** Hsp90 inhibitor would be a good candidate for the effective combined treatment with carbon ion radio-therapy.

**Fig. 1.**
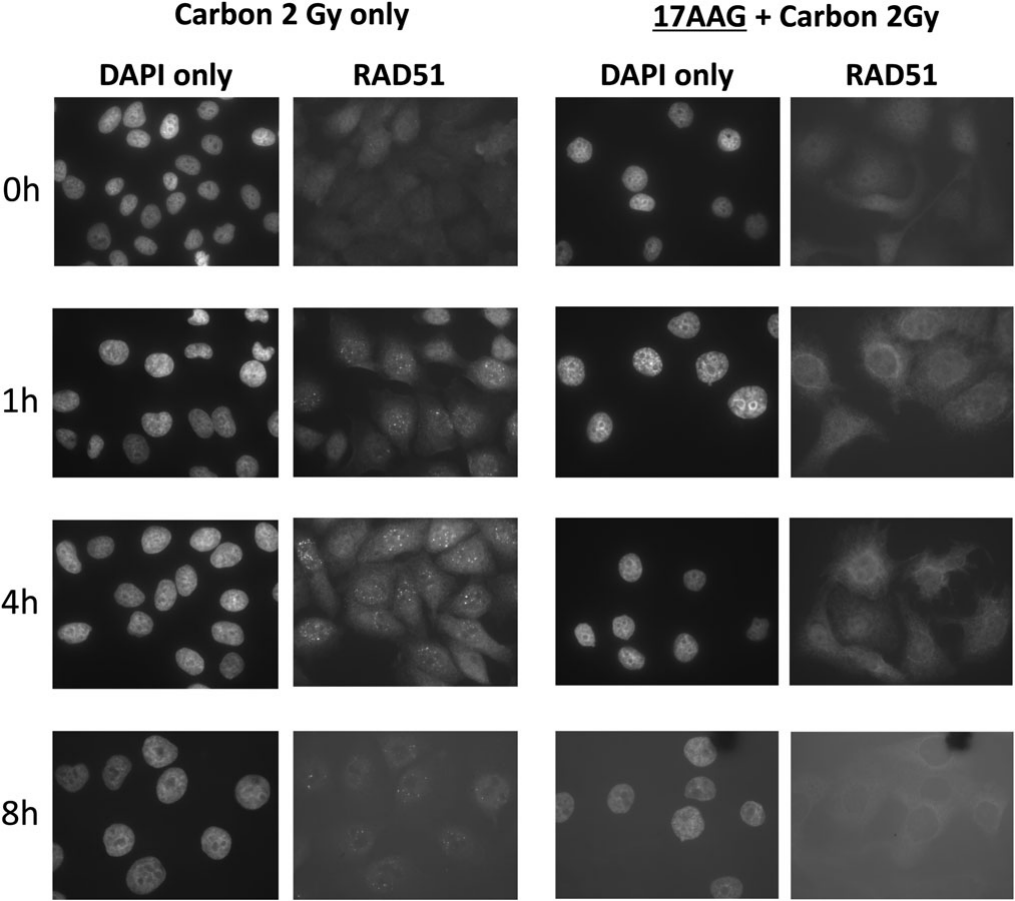
Post-irradiation Rad51 foci appearance kinetics in SQ5 cells irradiated with 2 Gy carbon ions compared with the combined treatment of 17AAG and carbon radiation (2 Gy).

Post-irradiation Rad51 foci appearance kinetics in SQ5 cells irradiated with 2 Gy carbon ions compared with the combined treatment of 17AAG and carbon radiation (2 Gy).
